# Does Treatment of Gingivitis During Pregnancy Improve Pregnancy Outcomes? A Systematic Review and Meta-Analysis

**DOI:** 10.3290/j.ohpd.b2183059

**Published:** 2021-10-22

**Authors:** Quynh-Anh Le, Guy D. Eslick, Kimberly Mathieu Coulton, Rahena Akhter, George Condous, Jörg Eberhard, Ralph Nanan

**Affiliations:** a Postgraduate Student, School of Dentistry and the Charles Perkins Center, Faculty of Medicine and Health, The University of Sydney, Sydney, New South Wales, Australia. Idea; methodological design; definition of search strategy; search and selection of articles; data extraction; qualitative analysis; wrote the manuscript.; b Professor, The Whiteley-Martin Research Centre, Discipline of Surgery, The University of Sydney, Nepean Hospital, New South Wales, Australia. Idea; methodological design; search and selection of articles; data extraction; synthesis of results; wrote the manuscript.; c Senior Lecturer, School of Dentistry, Faculty of Medicine and Health, The University of Sydney, Sydney, New South Wales, Australia. Methodological design; definition of search strategy; wrote the manuscript.; d Senior Lecturer, School of Dentistry, Faculty of Medicine and Health, The University of Sydney, Sydney, New South Wales, Australia. Methodological design; definition of search strategy; wrote the manuscript.; e Associate Professor, Acute Gynecology, Early Pregnancy, and Advanced Endoscopy Surgery Unit, Nepean Hospital, Sydney Medical School Nepean, University of Sydney, Sydney, Australia. Methodological design; definition of search strategy; wrote the manuscript.; f Professor, School of Dentistry and the Charles Perkin Center, Faculty of Medicine and Health, The University of Sydney, Sydney, New South Wales, Australia. Idea; methodological design; definition of search strategy; definition of search strategy; wrote the manuscript.; g Professor, Sydney Medical School Nepean and Charles Perkins Center Nepean, The University of Sydney, Sydney, New South Wales, Australia. Idea; methodological design; definition of search strategy; definition of search strategy; wrote the manuscript.

**Keywords:** birth weight, gingivitis, gingivitis treatment, preterm, randomised controlled trials

## Abstract

**Purpose:**

This study aimed to investigate whether treatment of gingivitis in pregnant women affects pregnancy outcomes.

**Materials and Methods:**

This was a systematic review and meta-analysis of clinical trials using PRISMA guidelines to appraise the treatment of gingivitis on pregnancy outcomes, including preterm birth (less than 37 weeks), low birth weight (less than 2,500 g), gestational age and birth weight. Pooled odds ratios (OR), mean difference, and 95% confidence intervals (CI) were calculated using the random effect model. A search was conducted in databases including Medline, Pubmed, Web of Science, Google Scholar and Embase without restrictions regarding language or date of publication.

**Results:**

Three clinical trials comprising 1,031 participants were included in this review. Treatment of gingivitis during pregnancy was associated with a decreased risk of preterm birth (OR = 0.44, 95% CI [0.20–0.98], P = 0.045) and higher birth weight (weighted mean difference (WMD) = 105.36 g, 95% CI [36.72–174.01], P = 0.003). Gestational age at birth in the treatment group (WMD = 0.31 weeks, 95% CI [–0.02–0.64], P = 0.64) as well as likelihood of low birth weight (OR = 0.92, 95% CI [0.38–2.21], P = 0.851) did not reach statistical significance.

**Conclusion:**

The results of this meta-analysis indicate that treatment of gingivitis in pregnancy may improve pregnancy outcomes including increased infants birth weight and reduced preterm births. Future trials are warranted to validate the true effect size of gingivitis treatment on pregnancy outcomes.

Recent epidemiological data report that more than 20 million infants worldwide (15.5% of all deliveries) are born with low birth weight and almost 11% of all live births are born premature.^13, 34^ Since low birth weight and prematurity are associated with high rates of neonatal mortality and morbidity, and the aetiology of both adverse pregnancy outcomes are complex, it is essential to manage known risk factors.^7^ Specifically, inflammatory response(s) during pregnancy have been associated with adverse gestational outcomes.^12^

Potential sources for maternal inflammation are periodontal diseases. Periodontal diseases result from infections of tooth supporting structures in response to bacterial accumulation.^11^ According to the severity of the disease, it could manifest as a reversible condition, such as gingivitis, or an irreversible form as periodontitis.^8, 11^ While periodontitis has been explored and shown to be associated with poor pregnancy outcomes, researchers have attempted to find out whether inflammation of the gingiva (gingivitis) which is curable and preventable could be related to adverse pregnancy outcomes.^18, 23, 25^ The systemic effects of gingivitis include but are not limited to bacteraemia and systemic dissemination of inflammatory mediators such as IL-1, IL-6 and TNF-α.^31^ During pregnancy, plaque-induced gingival inflammation is common and worsens by pregnancy-associated hormones.^6, 21, 35^ Despite this, treatment of gingivitis during pregnancy is often neglected by clinicians, as it is known that three months postpartum, gingival index significantly improves.^32^

Since gram-negative bacterium *Fusobacterium nucleatum*, which is commonly found in gingivitis, has been recognised as one of the most frequently isolated species from amniotic fluid cultures obtained from pregnant women with premature labour and intact placental membranes, suggesting a potential association between pregnancy-associated gingivitis and adverse pregnancy outcomes.^3, 17, 24, 29^ In conjunction with the high prevalence of gingivitis in pregnant women ranging between 60% to 75%, the prevention of gingivitis in pregnant women during pregnancy would provide enormous health benefits.^14^ Likewise, treatment of gingivitis is uncomplicated and accessible, which makes it more convenient for pregnant women to seek care if needed.

Therefore, the aim of this systematic review and meta-analysis is to evaluate the current evidence whether the treatment of gingivitis improves adverse birth outcomes in pregnant women.

## Materials and Methods

### Eligibility Criteria

Our proposed PICO question was ‘Does treatment of gingivitis compared to no treatment in pregnancy affect pregnancy outcomes?’ Therefore, studies were included in this review if they followed the inclusion criteria: (1) Study design was a clinical trial; (2) study population was pregnant women with gingivitis; (3) gingivitis treatment included sub-and supragingival cleaning and oral hygiene (OH) instructions or mouthwash; (4) the outcomes were preterm birth (less than 37 weeks), low birth weight (less than 2,500 g), gestational age and birth weight; (5) the data was presented such that an odds ratio (OR) and 95% confidence interval (CI) could be calculated. Studies that did not meet these criteria were excluded. There were no restrictions on language or years of publication.

### Literature Search

For the identification of eligible articles, two authors (QAL and GDE) conducted a systematic search of the Medline, PubMed, Google Scholar, Web of Science and Embase databases up to May 2020 following the (PRISMA) guidelines.^26^ Reference lists of relevant articles were also assessed. With non-English articles, we contacted the authors for the translation and original data. No search for unpublished literature or manual search was carried out.

An example of our search strategy involved: #1: (periodontal therapy) OR periodontal treatment) OR (scaling and root planing [MeSH Terms])) OR (supragingival and subgingival scaling [MeSH Terms])) OR mouthwash [MeSH Terms]) OR mouth rinse [MeSH Terms]; #2: (pregnant) OR gravida [MeSH Terms]) OR parturition [MeSH Terms]; #3: (gingivitis) OR gingival inflammation) OR pregnancy-gingivitis [MeSH Terms]; #4: (pregnancy outcome) OR preterm) OR low birthweight) OR prematurity) OR gestational age [MeSH Terms]) OR birth weight [MeSH Terms]) AND preterm low birth weight [MeSH Terms]; #1 AND #2 AND #3 AND #4.

The search was adapted depending on the databases using the same keywords and word combination.

### Study Selection

Two authors (QAL and GDE) screened title and abstracts of relevant records after removing duplication. Full-text publications were assessed from included abstracts. Studies were excluded if they reported subjects with periodontitis, study design was not clinical trial or no outcome of interest. Any disagreement was resolved between the two reviewers.

### Data Extraction

The data extraction was performed using a standardised extraction form, collecting information on the first author’s last name, publication year, study design, number of cases, number of controls, total sample size, country, continent, mean age, the risk of estimates or data used to calculate the risk estimates, CIs or data used to calculate the CI.

### Assessment of Quality of Selected Studies

Two authors (QAL and GDE) independently used the Cochrane risk assessment tool to evaluate the risk of bias consisting of random sequence generation, allocation concealment (selection bias), blinding of participants and personnel (performance bias), blinding of outcome assessment (detection bias), incomplete outcome data, selective reporting and other sources of bias.^15^ Each risk was determined as either of high, low or unclear risk of bias. Adjusted ratios were extracted in preference to non-adjusted ratios; however, where adjusted ratios were not provided, unadjusted ORs and CIs were calculated. Where more than one adjusted ratio was reported, the researchers chose the ratio with the highest number of adjusted variables. Where multiple risk estimates were available in the same study, for example, due to the use of different comparator groups, these were included as separate risk estimates. Inter-observer variability was evaluated by Kappa coefficient statistics.^20^ Any disagreement was resolved between two researchers.

### Synthesis of Results

Pooled odds ratios, mean difference, and 95% confidence intervals were calculated for outcomes of interest using a random-effects model.^9^ The degree of heterogeneity was calculated by using the I^2^ statistic, which represents the percentage of the total variability across studies. Publication bias was calculated using the Egger’s regression model.^16^ Statistical analyses were performed with the Comprehensive Meta-analysis package (Version 3.0, Biostat, Englewood, NJ (2014)).

## Results

### Study Selection

**Fig 1:**
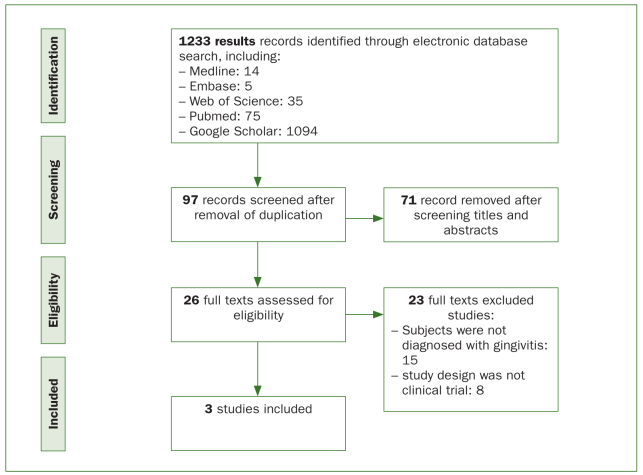
Flow diagram for the search and inclusion of eligible articles.

A total of 1,233 articles were identified using the search strategy described. There were 14 records retrieved from Medline, 5 records from Embase, 35 records from Web of Science, 75 articles from PubMed and 1,094 articles from Google Scholar. After eliminating duplication, 97 articles were preliminarily assessed by title and abstract screening. Seventy-one articles were removed, leaving 26 papers for a thorough full-text evaluation. Twenty-three references were eliminated because they did not meet the inclusion criteria and three full-text articles were eventually included in the analysis (Table 1). We contacted one author from Hungary for the translation and data enquiry.^28^ The PRISMA Flow diagram (Fig 1) summarises the literature search process and selection of studies.

**Table 1:** Study characteristics

No	Study	Country	Study design	Total sample size (n)	Participants	Mean age ± SD	Definition of gingivitis	Timing of treatment	Intervention and Control	Outcome Variables
1	Lopez 2005	Chile	Randomized controlled trial	870 – intervention group: n = 580 – control group: n = 290	Pregnant women with gingival inflammation with ≥ 25% sites with BOP and no sites with CAL >2 mm	– intervention group: 25.54±5.41 – control group: 24.98±4.55	≥ 25% sites with a positive sign of BOP and no sites with CAL >2 mm	Before 28 weeks of gestation	1. OH instructions, Supragingival and subgingival scaling, mouth rinse (0.12% chlorhexidine) daily 2. No treatment	PTB <37 weeks, LBW <2,500 g, PLBW, birthweight, gestational age
2	Novak 2018	Hungary	Randomized controlled trial	68 – intervention group: n = 33 – control group: n = 35	Pregnant women with gingivitis	– Intervention group: 28.6 ± 4.9 – control group: 27.8 ± 5.4	BOP present in ≥50% of sites and PD <4 mm in all sites measured	Between 24th and 37th week of gestation	1. Supra- and subgingival cleaning, OH instructions, polishing 2. No treatment	PTB <37 weeks, PTB< 32 weeks, LBW <2,500 g, LBW<1,500 g, birthweight, gestational age at birth
3	Kaur 2014	USA	Controlled clinical trial	120 – intervention group: n = 90 – control group: n = 30	Pregnant women between 16 to 24 weeks of gestation with gingivitis	– Intervention group: 23.1 ± 4.3 – control group: 21.7 ± 4.2	Gingival Index ≥2 at ≥50% of sites without periodontitis (clinical attachment loss >3 mm at ≥3 sites^1^	During pregnancy	1.OH instructions, supragingival and subgingival scaling 2.OH instructions	PTB<37 weeks LBW <2,500 g

BOP: bleeding on probing, PD: pocket depth; CAL: clinical attachment loss; OH: oral hygiene; PTB; preterm birth; LBW: low birth weight; PLBW: preterm low birth weight

### Study Characteristics

Three clinical trials were identified (2 randomised, and 1 non-randomised clinical trial) including 1,031 study participants.^19, 22, 28^ Pregnant women in the treatment group received supra-and subgingival scaling by instrumentation and OH instructions. In one study, pregnant women were provided a mouthwash (0.12% chlorhexidine) to rinse once a day until delivery.^22^ All participants in the control group were offered treatment after delivery. The diagnosis of gingivitis is depicted in Table 1. Lopez et al diagnosed gingivitis cases in case ≥ 25% of sites showed positive bleeding on probing and no sites with clinical attachment loss >2 mm.^22^ Novak et al selected gingivitis patients based on no periodontal probing depth ≥4 mm and bleeding on probing at ≥ 50% of the examined surfaces and Kaur et al identified gingivitis in patients with a gingival index equal or greater than 2 at ≥ 50% of the measured sites (Table 1).^19, 28^

### Outcomes

**Fig 2:**
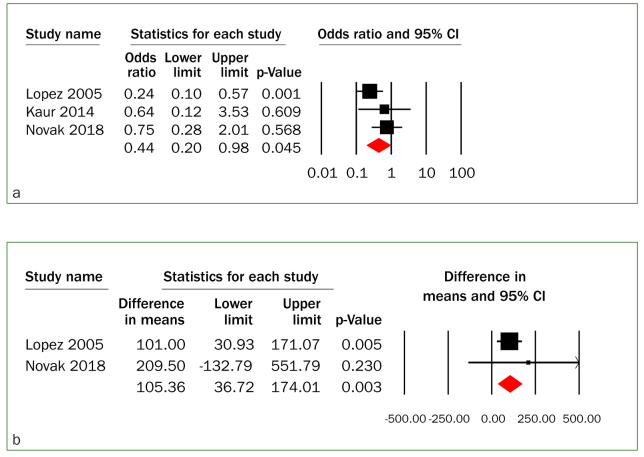
(a) Forest plot of summary crude odds ratios in the association between gingivitis treatment during pregnancy and preterm birth (PTB <37 weeks); and (b) differences in means of birth weight (gram) between gingivitis treatment and control group during pregnancy.

Gingivitis treatment in pregnant women resulted in a statistically significantly reduced risk for preterm birth (OR = 0.44, 95% CI [0.20–0.98] P = 0.045; I^2^ = 36.05, P = 0.21) (Fig 2a). There was a low level of heterogeneity and Egger’s regression analysis showed no evidence of publication bias (P = 0.67) (Fig 2). Women in the gingivitis treatment groups had a statistically significant higher birth weight (WMD = 105.36 grams, 95% CI [36.72–174.01], P = 0.003, I^2^ = 0.00, P = 0.54) (Fig 2b), and there was no evidence of heterogeneity.

However, the meta-analysis with randomised controlled trials showed that gingivitis treatment during pregnancy did not decrease risk of preterm birth (OR = 0.41, 95% CI [0.13–1.26], P = 0.12; I^2^ = 65.59, P = 0.09) or low birth weight (OR = 0.92, 95% CI [0.38–2.21], P = 0.851; I^2^ = 0.00, P = 0.61) compared to those who did not receive treatment. There was moderate to no evidence of heterogeneity, respectively. In addition, the gingivitis treatment group showed a non-statistically significant increase in gestational age at birth (WMD = 0.31 weeks, 95% CI [–0.02–0.64], P = 0.64; I^2^ = 8.26, P = 0.30). There was a low level of heterogeneity.

### Quality Assessment

The Cochrane risk of bias assessment of studies revealed that the two RCTs showed low levels of bias in five (sequence generation, allocation concealment, blinding of outcome assessment, incomplete outcome data and selective outcome data) out of seven domains. The non-randomised clinical trial showed low levels of risk of bias (blinding of outcome assessment, incomplete outcome data, selective outcome data), three high levels (sequence generation, allocation concealment and blinding of participants and personnel) of bias and one unclear (Table 2). The Kappa coefficient (K = 1) indicated the almost perfect agreement between two researchers.^20^

**Table 2a:** Risk of bias assessment according to Cochrane tool

Study and year of publication	Sequence generation (selection bias)	Allocation concealment (selection bias)	Blinding of participants and personnel (performance bias)	Blinding of outcome assessment (detection bias)	Incomplete outcome data (attrition bias)	Selective outcome data (reporting bias)	Other sources of bias
Lopez 2005	Low	Low	High	Low	Low	Low	Unclear
Novak 2018	Low	Low	High	Low	Low	Low	Unclear

**Table 2b:** Risk of bias assessment according to Cochrane tool

Study and year of publication	Bias due to confounding	Bias in selection of participants into the study	Bias in classification of interventions	Bias due to deviation from intended interventions	Bias due to missing data	Bias in measurement of outcomes	Bias in selection of the reported result
Kaur 2004	Low	Moderate	Low	Low	Low	Low	Low

## Discussion

This systematic review and meta-analysis of clinical trials provides evidence that treatment of gingivitis in pregnant women is associated with reduced risk of preterm birth and increased birth weight. The data also showed a trend between gingivitis therapy and reduced risk of low birth weight and increased gestational age; however, the results were not statistically significant. The treatment of gingivitis in pregnant women to improve birth outcomes is a global public health issue, especially when considering the high frequency of gingivitis in pregnant women and the ease of gingivitis treatment compared to the treatment of periodontitis.

This is the first systematic review and meta-analysis to assess the evidence regarding the effectiveness of gingivitis treatment and pregnancy outcomes in pregnant women. The majority of the studies identified in the search process explored the effect of periodontitis on pregnancy outcomes and did not separate periodontitis and gingivitis cases. There is substantial evidence of a decreased risk of perinatal mortality (RR = 0.53, 95%CI [0.30:0.93]), reduced risk of preterm birth (RR = 0.78, 95% CI [0.62–0.98]), and increased birthweight (WMD of 200.9 g, 95%CI [63.34–337.24]) in women who receive periodontitis treatment during pregnancy compared to periodontitis treatment after birth.^5^ Several biological mechanisms of how oral inflammatory processes including gingivitis and periodontitis, affect pregnancy outcomes can be relevant. First, the release of pro-inflammatory chemokines, like IL-1, IL-6, IL-8, TNF-α or PGE2 into the systemic bloodstream may reach the feta-placental unit and induce adverse pregnancy outcomes.^27, 30^ Specifically, interleukin-1 has been shown to induce preterm delivery.^27^ Furthermore, the induction of gingivitis in healthy individuals has been shown to increase acute systemic inflammation and associated levels of CRP, IL-6, MCP-1 and the activation of monocytes, which were reversible by adequate OH.^10^

Secondly, the translocation of oral pathogens, including anaerobic microorganisms might influence the integrity of the fetal–placental unit.^4^ In this context it is of interest that similarities have been described between the oral and placental microbiome.^1^ Interestingly, *Fusobacterium nucleatum*, a commonly found oral commensal, has been described in both amniotic fluids of women with potential premature delivery and in the placenta of women with chorioamnionitis, indicating a potential link between this bacterium and adverse pregnancy outcomes.^2, 4, 33^

Since methodological issues may affect the outcomes of this review, the Cochrane tool for the assessment of risk of bias was used to evaluate the selected studies. The assessment of risk of bias revealed that the three studies included were of moderate risk. The heterogeneity observed in the analysis may be due to differences in the diagnosis of gingivitis, the study population and the treatment applied.

 The strengths of this meta-analysis include the reasonably large sample size of 1,031 subjects from only three clinical trials. We believe that the comprehensive literature search also negates the likelihood that any important studies were missed. There were some limitations, including firstly the clinical trials could not blind the participants to the treatment they received. This is due to the nature of gingival treatment, which is invasive allowing no possibility to provide a placebo for a control group. The number of studies included was relatively small and features the need for more trials on this important matter.

## Conclusions

In conclusion, treatment of gingivitis during pregnancy was associated with a statistically significantly reduced risk of preterm birth and increased birth weight compared to no gingivitis treatment.
